# Case Report: SMARCA4-deficient uterine sarcoma with exceptional response to immune checkpoint inhibitor therapy

**DOI:** 10.3389/fonc.2026.1966486

**Published:** 2026-10-20

**Authors:** Qun Liu, Haili Kang, Jiao Wang, Haiyun Yu, Shuli Yang

**Affiliations:** 1 Department of Gynecological Oncology, Beijing Obstetrics and Gynecology Hospital, Capital Medical University, Beijing Maternal and Child Health Care Hospital, Beijing, China; 2 Laboratory for Clinical Medicine, Capital Medical University, Beijing, China; 3 Department of Pathology, Beijing Obstetrics and Gynecology Hospital, Capital Medical University, Beijing Maternal and Child Health Care Hospital, Beijing, China

**Keywords:** case report, immune checkpoint inhibitor, SMARCA4, SMARCA4-deficient uterine sarcoma, uterine sarcoma

## Abstract

**Background:**

SMARCA4-deficient uterine sarcoma (SDUS) is a rare and aggressive undifferentiated uterine mesenchymal malignancy characterized by inactivation of SMARCA4 gene leading to loss of nuclear SMARCA4/BRG1 immunohistochemical staining. Fewer than 40 cases have been reported worldwide since its first description in 2018, and the disease carries a median overall survival of only 7–9 months. SMARCA4 loss is observed across a spectrum of aggressive neoplasms, including smallcell carcinoma of the ovary, hypercalcemic type (SCCOHT), SMARCA4-deficient thoracic sarcoma, and SMARCA4-deficient nonsmallcell lung cancer, suggesting shared therapeutic vulnerabilities.

**Case:**

A 48-year-old woman presented with irregular vaginal bleeding for two years. Physical examination and magnetic resonance imaging (MRI) revealed a large pelvic mass centered in the uterus. The patient underwent total abdominal hysterectomy with bilateral salpingectomy, and pathological examination confirmed SDUS (stage IB, FIGO 2018). Genetic testing identified a somatic SMARCA4 splice-site variant (c.3951 + 2T>G). The tumor was TMB-low (0.06 Mut/Mb), PD-L1-negative (<1% by 22C3 pharmDx), microsatellite stable (MSS), and HLA-class-I heterozygous. Despite these conventional immunotherapy-unfavorable markers, adjuvant Gemcitabine-Docetaxel chemotherapy combined with pembrolizumab yielded a progression-free survival (PFS) of 23 months as of the last follow-up, far exceeding the previously reported median survival of SDUS.

**Conclusion:**

This case demonstrates that immune checkpoint inhibitors (ICI) may provide meaningful therapeutic benefit in selected patients with SDUS, including those lacking conventional predictive biomarkers (TMB-high, PD-L1 positivity, or MSI-H). While the observed 23-month PFS supports the hypothesis that SMARCA4 deficiency merits further investigation as a candidate molecular correlate of ICI responsiveness, definitive validation requires larger cohorts and ideally prospective, biomarker-driven studies. Definitive diagnosis relies on morphologic evaluation, loss of BRG1 protein expression by immunohistochemistry with retained INI-1, and detection of SMARCA4 gene alterations through genetic testing.

## Introduction

SMARCA4-deficient uterine sarcoma (SDUS) is a rare undifferentiated uterine mesenchymal malignancy characterized by loss of nuclear SMARCA4/BRG1 immunohistochemical staining. It was first described by Kolin in 2018 (1). Although extremely rare, SDUS is highly aggressive, with fewer than 40 cases reported worldwide to date and a median overall survival of only 7–9 months (2). SMARCA4 inactivation is shared by a family of SWI/SNF-deficient neoplasms that includes small cell carcinoma of the ovary, hypercalcemic type (SCCOHT) (3, 4), SMARCA4-deficient thoracic sarcoma (SMARCA4-DTS, recently renamed SMARCA4-deficient undifferentiated thoracic tumor, SMARCA4-UT) (5–7), and a subset of non-small-cell lung cancers (8, 9). Emerging evidence indicates that this shared molecular vulnerability may translate into overlapping therapeutic opportunities, most notably responses to ICI in patients whose tumors lack conventional predictive biomarkers such as PD-L1 expression or high tumor mutational burden (5, 10, 11). In this report, we present a rare case of SDUS with an exceptional response to ICI therapy and review the relevant literature to summarize its clinical presentation, diagnostic approach, differential diagnoses, treatment strategies, and the rationale for considering ICI beyond conventional biomarker selection. The clinical timeline of diagnosis, treatment and follow-up is shown in **Table 1**.

**Table 1:** Clinical timeline of diagnosis, treatment and follow-up.

Event	Key details
Initial presentation	48-year-old female presented with abnormal vaginal bleeding for 2 years. Pelvic ultrasound revealed a 6.1x6.9x5.5cm heterogeneous mass in the uterine cavity. MRI showed an 8.0x5.3x6.8cm mass with high DWI signal and uneven enhancement.
Surgical intervention	Underwent total abdominal hysterectomy with bilateral salpingectomy. Intraoperative findings: the uterus was enlarged to the size of a 12-week gestation, spherical and full, with soft consistency. The resected specimen showed gray-white masses in the uterine body and fundus, protruding into the uterine cavity and measuring approximately 6.5x6x5cm. The lesion invaded more than half of the myometrial thickness.
Pathological diagnosis	Final pathology: SMARCA4-deficient undifferentiated uterine sarcoma. Tumor size: 6.5×6×5cm. Immunohistochemistry: ER (-), PR (-), p53 (wild-type), Ki-67 (60%+), p16 (mostly +), SMA (+), BRG1 (-), Melan-A (+), INI-1 (+), Desmin (-), BCOR (-), Cyclin D1 (focal +), Myogenin (-), CD10 (-), TRK (-), HMB45 (-), CK (-).
Adjuvant therapy	Three cycles of chemotherapy with Gemcitabine and Docetaxel, followed by three cycles of pembrolizumab and subsequent pembrolizumab maintenance therapy.
First follow-up	Post-treatment evaluation at 3 months: no evidence of recurrence on pelvic CT and chest CT.
Latest follow-up	The patient remains recurrence-free with no immunotherapy-related adverse events (AEs), sustaining a progression-free survival (PFS) of 23 months as of the last evaluation.

## Medical history

A 48-year-old, gravida 2 para 1 (G2P1) woman presented with irregular vaginal bleeding persisting for two years. Her medical, surgical, family, and psychosocial history was unremarkable, with no personal or family history of malignancy and no tobacco use. She reported no prior exposure to pelvic radiation, hormonal therapy, or immunosuppression. Her Eastern Cooperative Oncology Group (ECOG) performance status was 1, and body mass index (BMI) was within the normal range. Baseline laboratory tests (complete blood count, comprehensive metabolic panel, coagulation profile, urinalysis) were within normal limits. Serum tumor markers, including cancer antigen 125 (CA-125: 18.4 U/mL), cancer antigen 19-9 (CA19-9: 12.6 U/mL), squamous cell carcinoma antigen (SCC-Ag: 0.9 ng/mL), and carcinoembryonic antigen (CEA: 2.1 ng/mL), were all within their reference ranges.

Physical examination: An approximately 4cm protruding, dark-red mass of medium consistency was observed at the external cervical os, it bled on palpation, with its base extending into the deep portion of the cervical canal. On bimanual examination, the uterus was enlarged to the size of an 12-week gestation, with a smooth, mobile contour, the bilateral adnexa were unremarkable, and inguinal and supraclavicular lymph node basins were clinically negative.

## Supportive examination

Cervical mass biopsy revealed an endometrial polyp at the lower uterine segment and internal cervical os. Ultrasound showed a space-occupying lesion in the uterine cavity measuring 6.1×6.9×5.5cm. Magnetic Resonance Imaging (MRI) revealed a mass-like lesion in the upper uterine cavity, measuring approximately 8.0×5.3×6.8cm, with isointense T1 and slightly hyperintense T2 signals, high DWI signal, and uneven moderate enhancement. The mass bulged into the myometrium with partially irregular margins, suggesting possible malignant invasion. The remainder of the uterine cavity contained multiple irregular cystic lesions with moderate to marked enhancement and low DWI signal, suggestive of polypoid lesions (**Figure 1**).

**Figure 1:**
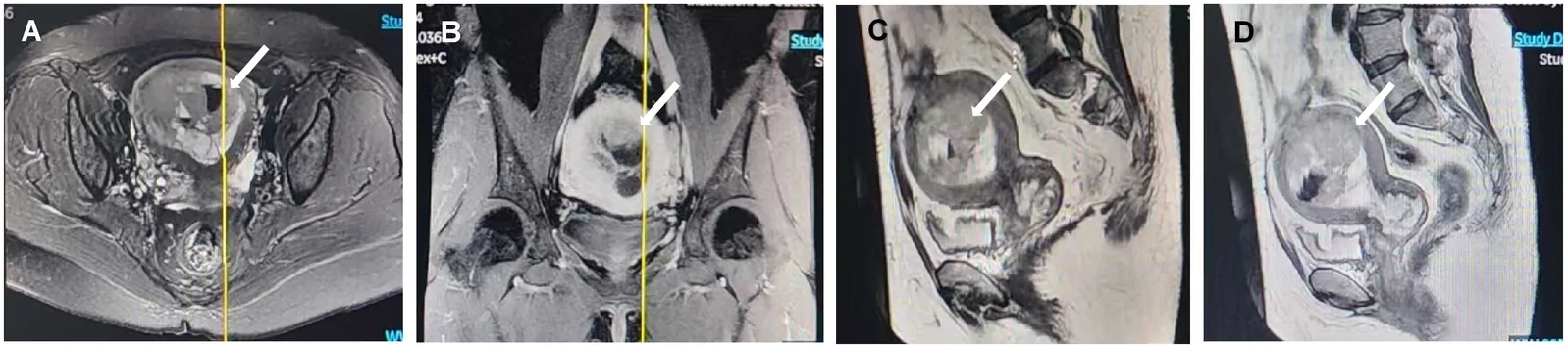
Axial **(A)**, coronal **(B)**, and sagittal **(C, D)** contrast-enhanced MRI images demonstrate a large, heterogeneous uterine mass (solid arrows). The lesion exhibits uneven moderate enhancement on diffusion-weighted imaging (DWI).

## Diagnosis and treatment

Although pathological examination of cervical biopsy specimens showed a polypoid lesion, the patient’s abnormal uterine bleeding and imaging findings suggested otherwise. Both ultrasound and MRI revealed a large intrauterine mass. The upper segment of the uterine cavity contained a highly vascularized lesion with high signal intensity on DWI, suggestive of malignant invasion into the myometrium. Therefore, total hysterectomy and bilateral salpingectomy were performed.

## Intraoperative and gross findings

Intraoperatively, the uterus was enlarged to the size of a 12-week gestation, spherical and full, with soft consistency. The bilateral fallopian tubes and ovaries appeared normal, with no palpable lymph node enlargement. The resected specimen showed gray-white masses in the uterine body and fundus, protruding into the uterine cavity and measuring approximately 6.5×6×5cm. The lesion invaded more than half of the myometrial thickness. The cut surface was gray-white, solid, and finely textured. Meanwhile multiple cystic-solid masses ranging from 0.5 to 2 cm in diameter fill the lower uterine segment and endocervical canal, and polypoid lesions are highly suspected.

## Pathological findings

Pathological Findings: Histopathology revealed a malignant uterine tumor with high-grade sarcomatous components and focal areas showing adenomatoid stromal differentiation. The tumor was predominantly composed of medium-to-large discohesive epithelioid cells with rhabdoid morphology, characterized by eccentrically placed nuclei, abundant eosinophilic cytoplasm, vesicular chromatin, prominent nucleoli, and frequent mitoses (approximately 15–20 mitoses per 10 high-power fields), with focal geographic necrosis and lymphovascular space invasion. Architecturally, the tumor grew in diffuse sheets interrupted by scattered leaf-like benign endometrial glands and a focal pseudo-phyllodes pattern. Histological and immunohistochemical findings confirmed a diagnosis of SMARCA4-deficient uterine sarcoma (**Figure 2**). The principal differential diagnoses considered included undifferentiated endometrial carcinoma (UDEC: positive for cytokeratin and claudin-4, typically wild-type SMARCA4), small cell carcinoma of the ovary, hypercalcemic type (SCCOHT: typically CD56, chromogranin A, synaptophysin and WT1 positive), dedifferentiated carcinoma, and high-grade endometrial stromal sarcoma; our case was rendered as SDUS based on the combination of predominant rhabdoid/epithelioid morphology, diffuse loss of SMARCA4 (BRG1) with retained INI-1 (SMARCB1), absence of cytokeratin/claudin-4 and SCCOHT-defining markers, and the identification of a somatic SMARCA4 splice-site variant.

**Figure 2:**
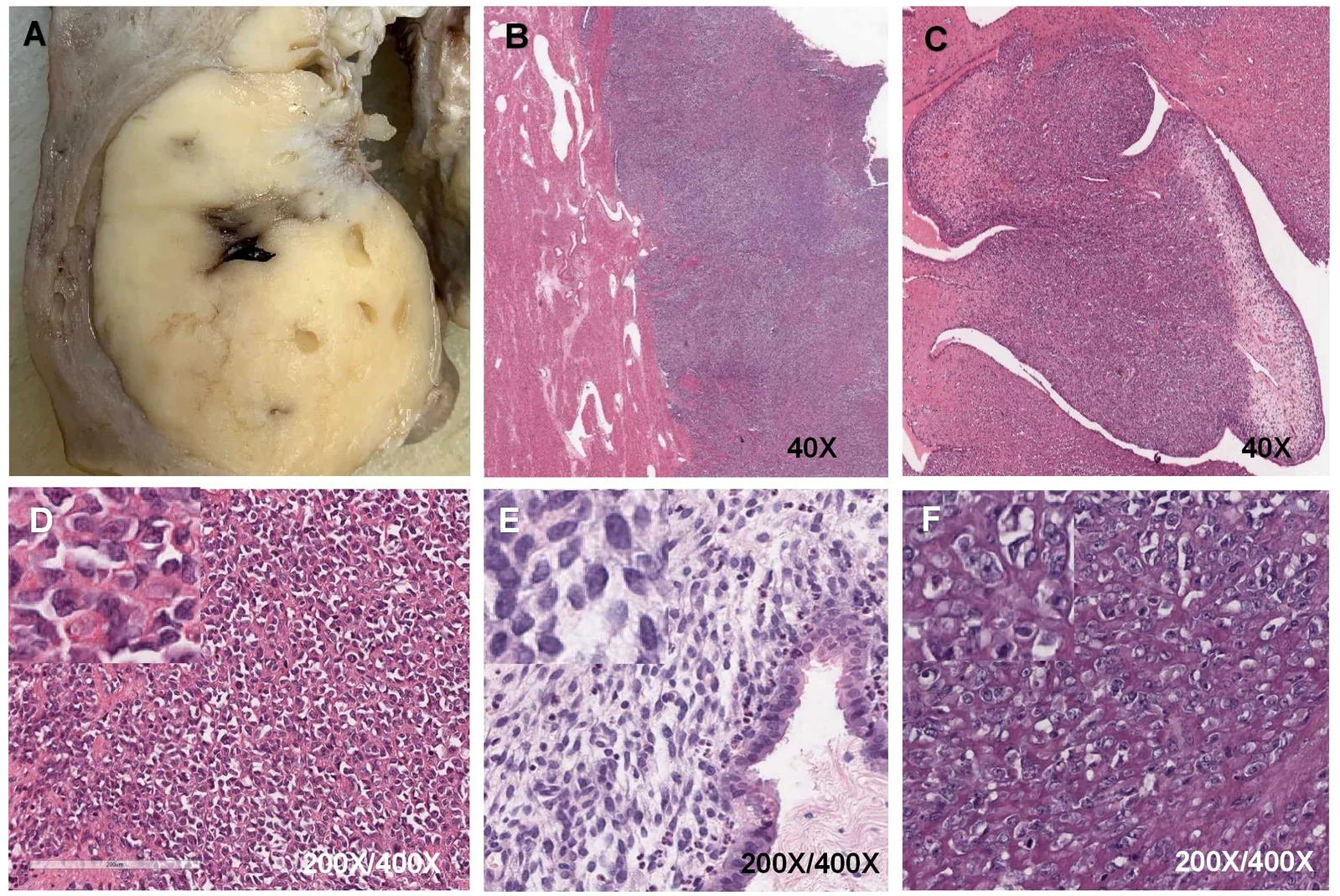
Histopathological findings. **(A)** Gross specimen showing a polypoid, cystic mass occupying the uterine cavity (no micro-scopic magnification). **(B, C)** Low-power H&E staining (40× magnification) demonstrating tumor cells arranged in nested, cord-like, and sheet-like patterns with abundant small-vessel stroma. Entrapped benign endometrial glands show dilated or slit-like morphology surrounded by cuff-like tumor infiltration. **(D–F)** Medium-power/high-power H&E views (200×/400× magnification) showing short-spindled to epithelioid tumor cells with moderate cellular pleomorphism, round-to-oval nuclei, prominent nucleoli, and frequent mitotic figures.

## Immunohistochemistry

Immunohistochemistry revealed: ER (-), PR (-), p53 (wild-type), Ki-67 (60%+), p16 (+,diffuse and strong), SMA (+), BRG1 (-), Melan-A (+), INI-1 (+), Desmin (-), BCOR (-), Cyclin D1 (focal +), Myogenin (-), CD10 (-), TRK (-), HMB45 (-), CK (-) (**Figure 3**).

**Figure 3:**
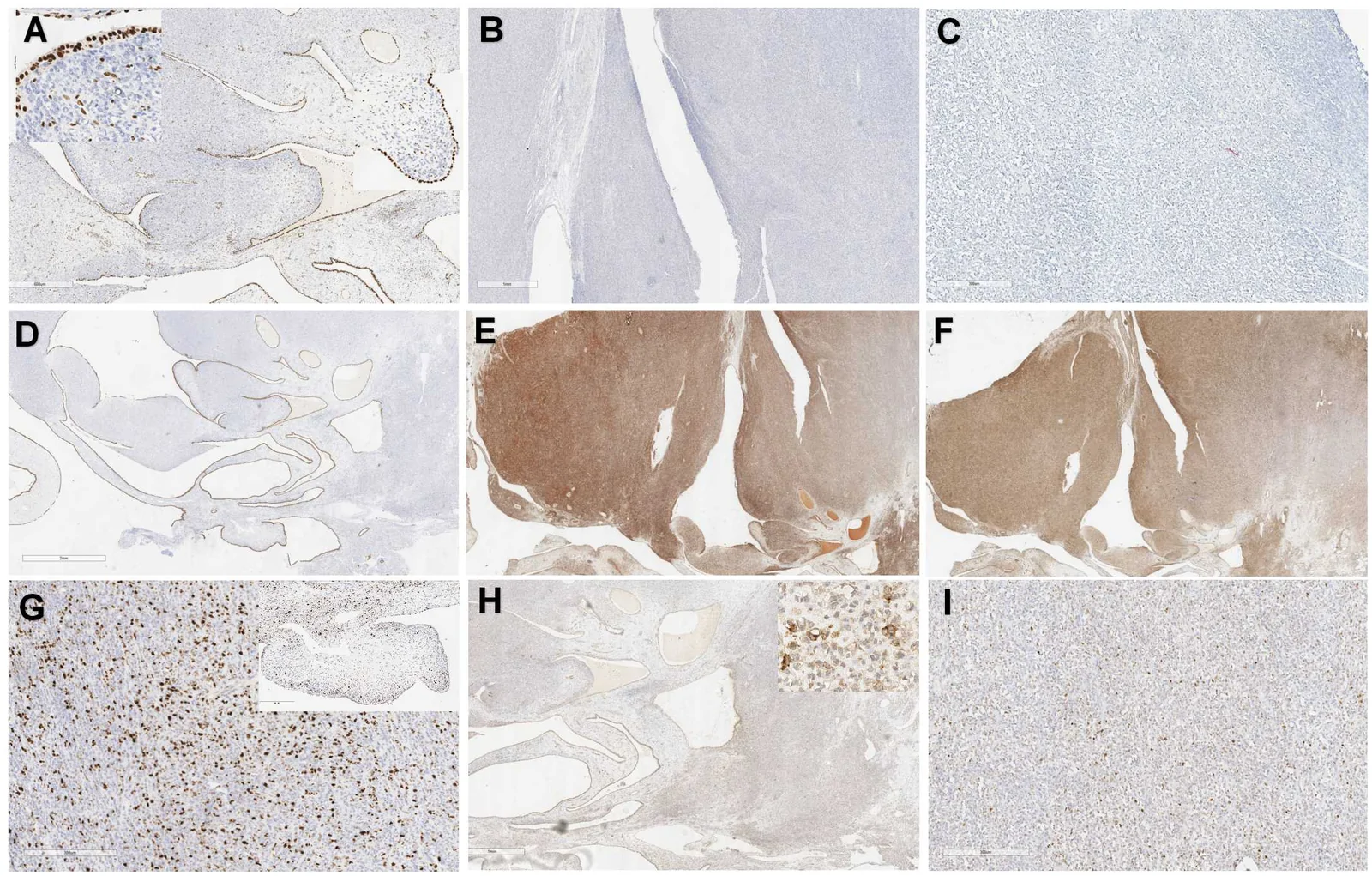
Immunohistochemical profile of the SDUS tumor (all images captured at 200× magnification). Tumor cells show loss of BRG1 **(A)**, and negative staining for ER **(B)**, PR **(C)**, and CK **(D)**, INI1 is retained **(E)**, SMA **(F)**, Ki-67 shows a high proliferative index **(G)**, CD10 **(H)**, p53 displays a wild-type staining pattern **(I)**.

## Genetic testing

Genetic testing identified a clinically significant somatic variant in *SMARCA4* (c.3951 + 2T>G). No pathogenic or likely pathogenic germline variants were identified. The tumor mutational burden (TMB) was 0.06 mutations/Mb, classified as TMB-Low. PD-L1 expression was negative. The tumor is microsatellite stablet (MSS). HLA class I typing showed heterozygosity.

## Postoperative diagnosis

SMARCA4-deficient uterine sarcoma, stage IB.

## Adjuvant treatment

Adjuvant therapy included three cycles of chemotherapy with Gemcitabine and Docetaxel, followed by three cycles of pembrolizumab and subsequent pembrolizumab maintenance therapy. Although the patient had PD-L1 negativity and low TMB, relevant literature has documented that such dissociation between conventional predictive biomarkers and ICI responsiveness has also been reported in SMARCA4-deficient neoplasms. Even in the setting of frequently low tumor mutational burden (TMB), these tumors may achieve favorable responses to ICI therapy (5, 8, 10). The patient tolerated adjuvant chemotherapy and immunotherapy well and is under regular surveillance.

## Follow-up

The patient remains recurrence-free with no immunotherapy-related adverse events (AEs), sustaining a progression-free survival (PFS) of 23 months as of the last evaluation.

## Informed consent and ethics

This case report was approved by the Ethics Committee of Beijing Obstetrics and Gynecology Hospital, Capital Medical University (retrospectively approved under the routine clinical case review protocol). Written informed consent was signed and obtained from the patient for the publication of de-identified clinical data, pathological photographs, laboratory results and treatment records in this manuscript. All personal identifiable information has been completely removed to protect patient privacy. The study was conducted in accordance with the principles outlined in the Declaration of Helsinki. The authors declare no conflicts of interest regarding the publication of this work.

## Discussion

The *SMARCA4* gene, located on chromosome 19p13, encodes BRG1, the catalytic ATPase subunit of the SWI/SNF chromatin remodeling complex, which regulates transcriptional activity through ATP-dependent nucleosome modification (12, 13). In gynecological malignancies, ARID1A/B mutations are frequently found in endometrial and ovarian cancers, whereas SMARCA4 alterations typically occur in non-epithelial tumors. Loss of SMARCA4 function disrupts chromatin remodeling, reduces chromosomal stability and has been associated with rhabdoid morphology (8, 13–15). SMARCA4 alterations are found in roughly 10% of non-small-cell lung cancers and are shared by a growing family of aggressive SWI/SNF-deficient neoplasms, including SCCOHT, SMARCA4-deficient undifferentiated thoracic tumor (SMARCA4-UT, also called SMARCA4-deficient thoracic sarcoma, SMARCA4-DTS) and SDUS (3, 5, 6, 8, 11).

According to data from The Cancer Genome Atlas (TCGA), SMARCA4 mutations occur in approximately 14.74% of endometrial cancers and 10.53% of uterine sarcomas. Within the female reproductive tract, SMARCA4 mutations are most consistently identified in SDUS, in a subset (20%) of undifferentiated and dedifferentiated endometrial carcinomas (UDEC/UDDEC) (16), and in small cell carcinoma of the ovary, hypercalcemic type (SCCOHT) (3). SDUS is the rarest of these entities, with fewer than 40 cases reported globally, and is highly aggressive with a very poor prognosis (2).

SDUS is an undifferentiated uterine mesenchymal malignancy first described by Kolin in 2018 (1) and often referred to as “malignant rhabdoid tumor of the uterus” because of its morphologic and immunophenotypic overlap with its ovarian counterpart (SCCOHT). The reported age at diagnosis ranges from 27 to 71 years, with a mean of approximately 36–49 years in published series (2, 12, 17). Due to its rarity, aggressiveness and absence of standardized therapy, the prognosis remains extremely poor (median overall survival 7–9 months) (2). Clinically, SDUS presents with non-specific symptoms, most commonly abnormal vaginal bleeding (18). Physical examination and imaging often reveal uterine or cervical masses. MRI may show long T2-weighted signals and a hypervascular mass, while CT is primarily used for assessing distant metastasis.

Histologically, SDUS grows in a diffuse, infiltrative pattern and frequently invades more than half of the myometrial thickness. The tumor is composed of medium-to-large discohesive epithelioid cells with rhabdoid morphology, abundant cytoplasm, moderate-to-marked nuclear atypia, prominent nucleoli and frequent mitotic activity. A leaf-like architecture of compressed, bland-appearing endometrial glands entrapped within the sarcoma is a characteristic (2, 19, 20). Lymphovascular space invasion is widespread or focal, and tumor necrosis is common (2, 19, 20). Loss of BRG1 (SMARCA4) protein expression by immunohistochemistry with preserved INI-1 (SMARCB1) staining is the diagnostic hallmark, molecular analysis typically reveals a SMARCA4 truncating or splice-site mutation, often accompanied by TP53, KRAS, STK11 and/or KEAP1 co-mutations (2, 16–18).

Important entities in the differential diagnosis are SCCOHT (3), which typically exhibits immunoreactivity for CD56, chromogranin A, synaptophysin and WT1, presents unilaterally in most cases, and is frequently associated with paraneoplastic hypercalcemia; UDEC, which demonstrates cytokeratin and claudin4 positivity, generally preserves SMARCA4 expression, and harbors PTEN/PIK3CA/TP53 mutations (16); and highgrade endometrial stromal sarcoma (21, 22). Lin et al. reported a patient with SDUS harboring a germline SMARCA4 mutation and a family history of SCCOHT (23), supporting SMARCA4 inactivation as a distinct oncogenic driver in SDUS. Germline SMARCA4 testing should therefore be offered to all patients with newly diagnosed SDUS to inform genetic counselling for the patient and at-risk family members.

SDUS is most often confused with SCCOHT because of overlapping morphology, immunophenotype (loss of BRG1 with retained INI-1) and underlying genetics (biallelic SMARCA4 inactivation) (3). Distinguishing features are, however, present: SCCOHT typically presents in young women as a large, unilateral adnexal mass, frequently with paraneoplastic hypercalcemia and is characteristically positive for CD56, chromogranin A, synaptophysin, cytokeratin and WT1 (3, 16), while SDUS arises in middle-aged women as a uterine/cervical mass with abnormal vaginal bleeding, is keratin-negative and usually WT1-negative (2, 16). Approximately 20% of UDEC cases show SMARCA4 (BRG1) loss, necessitating careful differential diagnosis (16, 21, 22). SDUS has a shorter median survival (~9 months) than UDEC (~36 months) (2, 16), presence of leaflike/phylloidiform architecture favors SDUS (16). UDEC frequently harbors TP53, PTEN and PIK3CA mutations with intact SMARCA4, whereas SDUS typically exhibits isolated SMARCA4 inactivation. Zheng et al. showed that all UDEC/DDEC tumors tested were diffusely SOX2-positive, while SDUS and SCCOHT were SOX2-negative (21). SOX2 is therefore a useful ancillary marker in this differential diagnosis.

SDUS has an extremely poor prognosis, with a median overall survival of 7–9 months even with active surgical treatment (2), and no established treatment guideline currently exists. For early-stage disease, total hysterectomy with bilateral salpingo-oophorectomy is the standard approach, ovarian preservation should be discussed with premenopausal patients with full informed consent regarding the uncertain malignant potential (2, 12). For advanced disease, reported systemic regimens include platinum-based chemotherapy, doxorubicin-ifosfamide and gemcitabine-taxane combinations, with the latter showing activity in selected patients (24–26). Gemcitabine-docetaxel, used in our case, has produced partial responses and transient disease stabilization in published case-series and small retrospective cohorts (25, 26), although durable benefit is uncommon. Preclinical studies suggest that SMARCA4 deficiency may sensitize cancer cells to Ataxia telangiectasia and Rad3-related inhibitors (ATRi) through synthetic lethality (27). EZH2 inhibitors have shown preliminary activity in SCCOHT and may also be relevant in SDUS given the reciprocal regulation between SWI/SNF and PRC2 (22, 28). CDK4/6 inhibitors are being explored in early-phase trials for SCCOHT and SMARCA4-deficient thoracic tumors (6, 29). Among SMARCA4-deficient sarcomas as a whole, pembrolizumab achieved an objective response rate of approximately 25% (3/12 patients, all partial responses) in the prospective AcSé pembrolizumab basket trial (5), and an individual-patient meta-analysis showed that 80.5% of SMARCA4-altered patients who received ICI derived some clinical benefit (partial response or stable disease), although the median PFS across the whole cohort was only 4.0 months (8). Whether these estimates apply directly to SDUS remains to be determined and is one of the questions our case was designed to inform.

Research on the immune microenvironment of SDUS is still limited. Gao et al. described prominent CD3+ and CD8+ tumor-infiltrating lymphocytes in an SDUS case (30), and Yao et al. demonstrated a highly immunogenic tumor immune microenvironment (TIME) with massive lymphocytic infiltration in SDUS (31). In the closely related SCCOHT, Jelinic et al. reported that patients responded to anti‐PD-1 therapy despite low tumor mutational burden, with strong PD-L1 expression and T-cell infiltration (3). Notably, such dissociation between conventional predictive biomarkers and ICI responsiveness has also been reported in SMARCA4-deficient neoplasms. Zhou et al. (11, 32) described durable clinical benefit from ICI in four out of five patients with thoracic SMARCA4-UT, all four of whom were PD-L1 negative. In a systematic review enrolling 160 patients with SMARCA4-altered malignancies, Ohta et al. (8) demonstrated that 80.5% of ICI-treated patients achieved partial response (PR) or stable disease (SD), even in the setting of frequently low tumor mutational burden (TMB). Multiple preclinical investigations have proposed potential underlying mechanisms. In ovarian cancer models, loss of SMARCA4 augments intrinsic tumor immunogenicity via upregulation of long terminal repeat (LTR) sequences, interferon-stimulated genes (ISGs), and components of the antigen presentation machinery (6, 8).

The multidisciplinary team’s decision to combine pembrolizumab with conventional chemotherapy in this patient warrants clear justification. Under most contemporary solid tumor guidelines, standard predictive biomarkers (low TMB, PD-L1 negativity, and MSS) would not support ICI administration. This therapeutic choice was grounded in multiple converging lines of evidence: PD-L1 status and TMB serve as unreliable predictors of ICI efficacy in SMARCA4-deficient neoplasms (6, 8, 11). Durable ICI responses have been reported in SCCOHT despite low TMB (3, 33), as well as in PD-L1-negative SMARCA4-deficient thoracic sarcoma (11). The AcSé pembrolizumab basket trial reported partial responses in 25% of patients with SMARCA4-deficient sarcoma or malignant rhabdoid tumor treated with single-agent pembrolizumab (5). Suzui et al. (10) reported the first case of ICI use in SDUS (pembrolizumab + lenvatinib) with disease stabilization; Gemcitabine and docetaxel both have documented immunogenic-cell-death effects that may synergize with PD-1 blockade. Given the absence of an established standard-of-care for SDUS and the patient’s informed consent, off-label addition of pembrolizumab was considered a reasonable compassionate option. The current case, with a 23-month PFS that markedly exceeds the historical median of 7–9 months, supports the wisdom of that decision while underscoring the need for prospective biomarker-driven trials.

The principal limitations of this report are inherent to its design as a single descriptive case. First, the contribution of pembrolizumab cannot be separated from that of the concurrent Gemcitabine-Docetaxel chemotherapy, whether the response would have been as durable with chemotherapy alone is not known. Second, although durable responses to ICI have been observed in SMARCA4-deficient thoracic tumors and SCCOHT, the cross-trial median PFS after ICI in SMARCA4-altered malignancies is approximately 4 months (8) and hyper-progression has been reported in SMARCA4-deficient small-cell lung cancer (6, 8), hence a single case cannot establish ICI efficacy in SDUS, further rigorous validation in additional cases will be essential to establish the therapeutic utility of ICI. Third, comprehensive tumor-immune-microenvironment data (TIL counts, multiplex immunofluorescence, single-cell RNA sequencing) are not available for this case, limiting our ability to test specific mechanistic hypotheses for the observed response. Fourth, the relatively short follow-up (23 months) is encouraging but does not preclude late recurrence. Fifth, the patient experienced a two-year history of intermittent vaginal bleeding before diagnosis, raising the possibility that her tumor may have pursued a more indolent course than is typical for SDUS, this prolonged pre-diagnostic interval may itself bias PFS estimates favorably and should be acknowledged as a potential confounder when comparing her outcome to historical series with predominantly short symptom-to-diagnosis intervals.

## Conclusion

We report a rare case of SDUS in a 48-year-old woman, diagnosed by loss of BRG1 expression on immunohistochemistry and a somatic SMARCA4 splice-site variant (c.3951 + 2T>G) on genetic testing. Despite immunotherapy-unfavorable biomarkers (TMB-low, PD-L1-negative, MSS), the patient achieved a 23-month PFS following Gemcitabine–Docetaxel chemotherapy combined with pembrolizumab, far exceeding the reported median OS of 7–9 months. These observations, together with prior cross-tumour evidence, generate the hypothesis that SMARCA4 deficiency may serve as a candidate molecular correlate of ICI responsiveness across the SDUS, SCCOHT, SMARCA4-deficient thoracic tumor spectrum (5, 6, 8, 10, 11) and provide additional rationale for prospective, biomarker-stratified evaluation of immune checkpoint inhibitors in SDUS. As the evidence currently rests on a small number of cases and across heterogeneous tumor types, broader conclusions regarding routine off-label ICI use in SDUS await prospective, biomarker-stratified clinical trials.

## Patient perspective

The patient provided written informed consent for the publication of this case report and accompanying images. Following treatment, the patient expressed satisfaction with her clinical outcome and willingness to share her experience to help future patients diagnosed with this rare disease. She emphasized the importance of early diagnosis and timely initiation of comprehensive treatment for rare gynecologic malignancies.

## Data Availability

The original contributions presented in the study are included in the article/supplementary material. Further inquiries can be directed to the corresponding authors.
